# Decoding Cervical Neuroendocrine Carcinoma: A Practical Review of Diagnostic Pitfalls, Differential Diagnosis and Molecular Insights

**DOI:** 10.3390/diagnostics16121781

**Published:** 2026-06-09

**Authors:** Andreea Onofrei (Popa), Gabriela Gurau, Gabriela Patrichi, Alina-Mihaela Gurau, Roxana-Cristina Mehedinti, Catalin-Bogdan Satala

**Affiliations:** 1 Faculty of Medicine and Pharmacy, Medical and Pharmaceutical Research Center, “Dunărea de Jos” University of Galati, 800008 Galati, Romania; andreea.onofrei@ugal.ro (A.O.); gabriela.gurau@ugal.ro (G.G.); roxana.mehedinti@ugal.ro (R.-C.M.); catalin.satala@ugal.ro (C.-B.S.); 2 The School for Doctoral Studies in Biomedical Sciences, “Dunărea de Jos” University of Galati, 800008 Galati, Romania; alina.gurau@ugal.ro; 3 “Sf. Ioan” Clinical Emergency Pediatric Hospital, 800487 Galati, Romania; 4 The Doctoral School of Medicine and Pharmacy, “George Emil Palade” University of Medicine, Pharmacy, Science and Technology, 540142 Targu Mures, Romania; 5 Department of Pathology, Clinical County Emergency Hospital Braila, 810325 Braila, Romania

**Keywords:** cervical neuroendocrine carcinoma, small cell neuroendocrine carcinoma (SCNEC), large cell neuroendocrine carcinoma (LCNEC), immunohistochemistry, diagnostic pitfalls

## Abstract

Cervical neuroendocrine carcinoma (NECC) is a rare but highly aggressive cervical malignancy, accounting for approximately 1% of invasive cervical cancers. Diagnosis is challenging because NECC overlaps morphologically with other poorly differentiated cervical and metastatic tumors, may show crush artifact in small biopsies, and can display variable expression of conventional neuroendocrine markers. This narrative review provides a practical synthesis of the histopathological, immunohistochemical, molecular, and clinical features of NECC, with emphasis on diagnostic pitfalls and differential diagnosis. Recent English-language literature on cervical neuroendocrine carcinoma was reviewed and qualitatively integrated, focusing on morphology, immunohistochemistry, HPV association, molecular alterations, prognosis, and management-relevant diagnostic issues. Accurate diagnosis requires integration of morphology, epithelial differentiation, neuroendocrine marker expression, HPV-related context, and clinic-radiological correlation. Useful markers include broad-spectrum cytokeratins, synaptophysin, chromogranin A, CD56, INSM1, p16, and Ki-67, with additional markers selected according to the differential diagnosis. Diffuse block-type p16 expression and high-risk HPV detection support a cervical HPV-associated origin but are not specific for neuroendocrine differentiation. Molecular studies show frequent association with HPV18 and recurrent alterations involving PI3K/AKT, RAS/MAPK, and TP53-related pathways, although these findings remain insufficiently validated for routine prognostic or therapeutic stratification. NECC requires early recognition and a multimodal diagnostic approach because of its aggressive behavior and poor prognosis. A practical, stepwise integration of morphology, immunohistochemistry, molecular findings, and clinical–radiological data may improve diagnostic consistency and support multidisciplinary management.

## 1. Introduction

Neuroendocrine neoplasms (NENs) comprise a heterogeneous group of tumors characterized by neuroendocrine differentiation, reflected by the expression of neuroendocrine markers and, in some cases, by the capacity to produce peptide hormones or biogenic amines. According to their site of origin, morphology, and degree of differentiation, NENs encompass a broad spectrum of entities, including well-differentiated neuroendocrine tumors (NETs) and poorly differentiated neuroendocrine carcinomas (NECs) [1]. From a histogenetic perspective, NETs and NECs are epithelial neuroendocrine neoplasms and typically express cytokeratins, a feature that is diagnostically important in distinguishing them from non-epithelial tumors that may enter the differential diagnosis [1,2].

Although NENs arise most commonly in the gastrointestinal tract, pancreas, and lung, they may rarely occur in the female genital tract, including the uterine cervix. Neuroendocrine carcinoma of the cervix (NECC) is an uncommon but highly aggressive subtype of cervical malignancy, accounting for approximately 0.9–1.5% of invasive cervical cancers in most published institutional series, registry-based analyses, and systematic reviews [3]. These estimates are derived mainly from retrospective cohorts from North America, Europe, and East Asia, and reported frequencies may vary according to geographic region, diagnostic criteria, referral patterns, and case ascertainment [1,3]. Among cervical neuroendocrine neoplasms, small cell neuroendocrine carcinoma (SCNEC) is the most frequent subtype, whereas large cell neuroendocrine carcinoma (LCNEC) is less common, and well-differentiated neuroendocrine tumors are exceptionally rare in this anatomical site. Clinically, NECC usually affects adult women, with a broad reported age range and many cases diagnosed in mid-adulthood. Presenting symptoms are generally non-specific and overlap with those of other cervical malignancies, including abnormal vaginal bleeding, postcoital bleeding, pelvic pain, or vaginal discharge. Cervical cytology may reveal malignant or high-grade atypical cells, but Pap smear findings are not sufficiently specific for NECC; therefore, definitive diagnosis requires histopathological assessment supported by immunohistochemistry and clinico-radiological correlation [4,5,6].

The clinical relevance of NECC extends beyond its rarity. In comparison with the more common histological subtypes of cervical cancer, such as squamous cell carcinoma and adenocarcinoma, NECC is associated with a markedly more aggressive clinical course, including a higher rate of lymphovascular invasion, early lymph node involvement, metastasis at presentation or early during follow-up, frequent distant recurrence, and a substantially worse overall prognosis. Reported survival outcomes remain poor even in patients diagnosed at relatively early stages, emphasizing the importance of prompt recognition and accurate pathological classification [7].

From a diagnostic standpoint, NECC represents a particularly challenging entity. Histologically, these tumors may overlap with other poorly differentiated cervical malignancies, especially poorly differentiated squamous cell carcinoma, high-grade adenocarcinoma, lymphoma, sarcoma, melanoma, and other small round blue cell tumors [8]. In limited biopsy samples, the diagnosis may be further complicated by crush artifact, tumor necrosis, and sampling bias, while mixed tumors containing neuroendocrine and non-neuroendocrine components may be only partially represented [9]. In addition, the distinction between a primary cervical neuroendocrine carcinoma and metastatic neuroendocrine carcinoma involving the cervix may be difficult and requires careful integration of morphology, immunohistochemistry, and clinico-radiological findings.

Immunohistochemistry plays a central role in establishing the diagnosis. Conventional neuroendocrine markers, including synaptophysin, chromogranin A, CD56, and neuron-specific enolase, remain widely used in routine practice, although their sensitivity and specificity are variable. Synaptophysin and CD56 are generally regarded as more sensitive markers, whereas chromogranin A is more specific but less consistently expressed in poorly differentiated tumors [10,11]. More recently, insulinoma-associated protein 1 (INSM1) has emerged as a highly sensitive marker in high-grade neuroendocrine neoplasms and may be particularly useful in diagnostically challenging cases. At the same time, confirmation of epithelial differentiation with keratin markers is essential, especially in small biopsy specimens and in tumors with ambiguous morphology. Importantly, no single immunohistochemical marker is sufficient to confirm NECC or determine the primary site in isolation [12,13].

Another important aspect of NECC is its strong association with high-risk human papillomavirus (HPV), particularly HPV18 and, less frequently, HPV16 [14]. This link supports a cervical origin in most cases and provides biological continuity with other HPV-related cervical malignancies [15]. At the molecular level, recent studies have identified recurrent alterations involving pathways such as PI3K/AKT, RAS/MAPK, and TP53, as well as HPV integration-related genomic events, suggesting that NECC has a distinct pathogenetic profile with potential diagnostic, prognostic, and therapeutic implications [16,17,18,19]. However, most molecular data are derived from small retrospective cohorts and remain insufficiently validated for routine prognostic or therapeutic stratification [16,17,18,19].

Despite increasing recognition of this entity, major challenges remain in daily practice. The rarity of NECC has limited the development of robust prospective studies and has contributed to persistent uncertainty regarding diagnostic criteria, prognostic stratification, and optimal management. Consequently, much of the available evidence derives from small retrospective series, case reports, and extrapolation from neuroendocrine carcinomas arising in other organs, particularly the lung.

In this context, a focused and updated review of the diagnostic landscape of NECC is warranted. The aim of the present narrative review is to summarize the current evidence regarding the histopathological, immunohistochemical, and molecular features of NECC, with particular emphasis on diagnostic pitfalls, differential diagnosis, and the practical integration of ancillary markers that may support accurate classification in routine pathology. By integrating morphology, immunohistochemistry, HPV-related findings, molecular data, and clinically relevant diagnostic scenarios, this review seeks to provide a practical framework for pathologists and gynecologic oncologists faced with this rare but aggressive tumor.

## 2. Materials and Methods

This study was designed as a narrative review aimed at summarizing and critically integrating the available evidence on the diagnostic, histopathological, immunohistochemical, molecular, prognostic, and clinically relevant features of neuroendocrine carcinoma of the cervix (NECC), with particular emphasis on diagnostic pitfalls, differential diagnosis, and practical issues encountered in routine pathology.

Relevant English-language publications were identified through targeted searches of PubMed, Scopus, and Web of Science, supplemented by manual review of reference lists from key articles. The search focused mainly on literature published between January 2016 and March 2026, although selected earlier landmark publications were considered when relevant for classification, epidemiology, or clinical context. Search terms included combinations of “cervical neuroendocrine carcinoma”, “small cell neuroendocrine carcinoma cervix”, “large cell neuroendocrine carcinoma cervix”, “immunohistochemistry”, “INSM1”, “HPV”, “molecular alterations”, “next-generation sequencing”, “diagnostic pitfalls”, “prognosis”, and “treatment”.

Publications were considered when they provided relevant information on morphology, immunohistochemistry, HPV association, molecular alterations, differential diagnosis, prognosis, survival, or treatment-related aspects of cervical neuroendocrine neoplasms. Because this was a narrative review rather than a formal systematic review, no PRISMA flow diagram, formal risk-of-bias scoring system, or quantitative meta-analysis was performed.

To reduce selection bias and improve transparency, the review was organized around predefined thematic objectives: histopathological spectrum, immunohistochemical diagnosis, HPV-related and molecular findings, diagnostic pitfalls, differential diagnosis, prognostic factors, therapeutic considerations, and future research directions. Evidence derived from small retrospective cohorts, case reports, or isolated therapeutic observations was interpreted cautiously and treated as preliminary or hypothesis-generating when appropriate.

## 3. HPV-Related and Molecular Pathogenesis of Cervical Neuroendocrine Carcinoma

High-risk human papillomavirus (HPV) plays a central role in the pathogenesis of most cervical neuroendocrine carcinomas, particularly small cell neuroendocrine carcinoma (SCNEC) [20]. Similar to other HPV-related cervical malignancies, NECC is strongly associated with oncogenic viral infection; however, compared with squamous cell carcinoma and usual-type adenocarcinoma, this tumor subtype shows a particularly strong association with HPV18, followed by HPV16. This distribution suggests that NECC may represent a biologically distinct form of HPV-driven malignancy, with specific molecular and clinicopathological characteristics [21,22].

The demonstration of high-risk HPV is not only pathogenetically relevant but may also support the diagnosis of a primary cervical tumor, especially in cases in which the distinction from metastatic neuroendocrine carcinoma is clinically or morphologically challenging [23,24]. Several methods have been used for HPV detection in this setting, including polymerase chain reaction for viral genotyping, in situ hybridization for tissue-based localization, and, more recently, next-generation sequencing approaches that allow a more detailed assessment of viral integration events within the host genome [25].

Emerging molecular evidence indicates that HPV integration is not merely an epiphenomenon, but may contribute directly to tumor progression through genomic instability and dysregulation of cancer-related genes. Distinct integration profiles have been reported for HPV18 and HPV16 in cervical small cell neuroendocrine carcinoma. HPV18 integration has been described at chromosomal loci such as 8q24.21 and 14q13.2, including gene-rich regions with potential downstream effects on cancer-related genes such as MYC and PVT1. MYC is a major transcriptional regulator involved in cell-cycle progression, cellular growth, metabolism, and genomic instability, whereas PVT1 is a long non-coding RNA located at 8q24 that may cooperate with MYC-driven oncogenic programs. In contrast, HPV16 integration has been identified at loci such as 20q11.21 and 17q12, in proximity to genes including NR4A2 and PGAP3. NR4A2 encodes a nuclear receptor involved in transcriptional regulation, differentiation, and cell survival, whereas PGAP3 participates in glycosylphosphatidylinositol-anchor remodeling; however, the functional relevance of these genes in NECC remains less clearly validated than that of MYC-associated alterations [25,26]. These findings suggest that different HPV subtypes may promote tumorigenesis through partially distinct genomic mechanisms [27,28].

Beyond HPV-related events, recent advances in next-generation sequencing have expanded the understanding of the molecular landscape of NECC, although most available studies remain retrospective and involve small or moderately sized cohorts. Targeted NGS and whole-exome sequencing studies have reported recurrent alterations in pathways involved in cell proliferation, survival, genomic stability, and invasion. Published cohorts include targeted sequencing of 51 patients with SCNEC, molecular profiling of 62 high-grade NECC specimens, WES-based comparative genomic analysis in NECC, and a larger registry-based analysis of 109 women with high-grade NECC who underwent tumor-based molecular testing [28,29,30,31].

Across these studies, the most frequently reported alterations involve PI3K/AKT/mTOR signaling, RAS/MAPK signaling, TP53-related pathways, MYC-family alterations, and, less commonly, RB1, PTEN, KIT, CTNNB1, and other potentially targetable genes. Alterations involving the PI3K/AKT signaling pathway appear to be among the most relevant molecular events identified to date. Activating alterations in the PI3K/AKT/mTOR pathway are among the most consistently reported molecular events in NECC. PIK3CA alterations have been identified across several cohorts, with reported frequencies ranging from approximately 10% to 27%, depending on cohort size, histological inclusion criteria, and sequencing platform. PTEN alterations or loss have also been reported, further supporting the involvement of this pathway in tumor development and progression [16,27,31]. Alterations involving the RAS/MAPK pathway have also been described, including KRAS mutations and, in some cohorts, GNAS alterations. However, reported frequencies vary substantially across studies and should be interpreted cautiously because of small sample sizes and heterogeneous testing methods [30,31]. These observations are of potential clinical interest because selected alterations may be therapeutically actionable in recurrent or metastatic disease. However, evidence supporting targeted therapy in NECC remains limited to small molecular cohorts and isolated clinical observations; therefore, molecularly guided treatment should currently be regarded as investigational or individualized rather than standard of care.

Abnormalities in tumor suppressor and transcriptional regulatory pathways may also contribute to the aggressive behavior of these tumors. TP53 alterations are biologically relevant because they impair DNA-damage response, apoptosis, and cell-cycle control, although their prognostic value in NECC remains inconsistently validated across cohorts. RB1 alterations appear less frequent than in pulmonary small cell carcinoma, but registry-based data suggest that RB1-altered high-grade NECC may be associated with shorter overall survival. Additional reported alterations include genes involved in transcriptional regulation and lineage plasticity, such as BCL6, NCOA3, and SOX2. BCL6 is a transcriptional repressor implicated in cell survival and differentiation programs, NCOA3 functions as a nuclear receptor coactivator with roles in hormone-related and growth-factor signaling, and SOX2 is linked to stemness, squamous/neuroendocrine lineage plasticity, and aggressive tumor behavior. Nevertheless, the clinical significance of these alterations in NECC remains exploratory and requires validation [4,23].

Data comparing paired primary and metastatic NECC specimens from the same patient remain scarce. Some molecular series have included tumor tissue obtained from either primary or metastatic sites and at different disease time points, but systematic paired primary-metastatic WGS, RNA-seq, or single-cell analyses have not yet been established as a robust evidence base for NECC. As a result, the clonal evolution of NECC, including acquisition of private metastatic mutations, therapy-selected subclones, and transcriptional changes associated with dissemination, remains poorly defined. Future paired analyses of primary, recurrent, and metastatic lesions could clarify whether progression is driven by additional alterations in DNA repair, PI3K/AKT, RAS/MAPK signaling, epithelial–mesenchymal transition, immune evasion, or neuroendocrine lineage plasticity. Table 1 summarizes the main molecular profiling studies of cervical neuroendocrine carcinoma, including sequencing methods, cohort sizes, recurrently altered genes, reported frequencies, and key limitations.

In addition to coding genomic alterations, non-coding RNAs have emerged as potentially important regulators of tumor behavior. Several microRNAs, including let-7c, miR-100, miR-125b, miR-143, miR-145, and miR-199a-5p, have been reported to be downregulated in advanced-stage cervical SCNEC compared with early-stage disease. Reduced expression of many of these microRNAs has also been associated with lymph node metastasis and poorer survival, suggesting a possible role in tumor progression and metastatic dissemination [16,31]. However, these findings remain exploratory, and microRNA profiles are not currently used for routine diagnostic, prognostic, or therapeutic decision-making in NECC.

Taken together, the currently available evidence indicates that NECC is driven by a complex interplay between high-risk HPV infection and recurrent molecular alterations affecting major oncogenic and tumor suppressor pathways. The most reproducible findings involve HPV18 association, HPV integration-related genomic disruption, PI3K/AKT/mTOR pathway alterations, RAS/MAPK pathway alterations, and TP53-related pathway abnormalities. However, most molecular studies remain limited by small sample size, retrospective design, heterogeneous sequencing platforms, and incomplete clinical annotation. At present, molecular findings are best regarded as biologically informative and potentially useful for selected recurrent or metastatic cases, but they have not yet been validated as stand-alone diagnostic, prognostic, or therapeutic determinants in routine NECC management.

## 4. Histopathological Features of Cervical Neuroendocrine Neoplasms

Cervical neuroendocrine neoplasms comprise a rare and morphologically diverse group of tumors that includes exceptionally uncommon well-differentiated neuroendocrine tumors and, far more frequently, poorly differentiated neuroendocrine carcinomas. In routine practice, the greatest diagnostic relevance lies in the recognition of high-grade neuroendocrine carcinomas, particularly small cell neuroendocrine carcinoma (SCNEC) and large cell neuroendocrine carcinoma (LCNEC), because these entities are associated with highly aggressive biological behavior and require prompt clinicopathological correlation.

### 4.1. Well-Differentiated Neuroendocrine Tumors of the Cervix

Well-differentiated neuroendocrine tumors of the cervix are exceedingly rare. When present, they generally display architectural patterns similar to neuroendocrine tumors arising in other anatomical sites, including trabecular, nested, ribbon-like, insular, or glandular arrangements. The neoplastic cells are usually relatively uniform and may be cuboidal, polygonal, or columnar, with moderate amounts of eosinophilic or amphophilic cytoplasm [32].

Nuclear morphology varies according to tumor grade. Low-grade lesions usually show round to oval nuclei, finely granular chromatin, inconspicuous nucleoli, low mitotic activity, and absent or limited necrosis. Because well-differentiated cervical neuroendocrine tumors are exceptionally rare, most diagnostic and clinical discussions focus on poorly differentiated neuroendocrine carcinomas, particularly SCNEC and LCNEC [32,33,34].

### 4.2. Small Cell Neuroendocrine Carcinoma

SCNEC is the most common NECC and represents the prototypical high-grade neuroendocrine malignancy at this site. Histologically, SCNEC is composed of sheets, nests, cords, or trabeculae of small to intermediate-sized tumor cells with scant cytoplasm, resulting in a high nuclear-to-cytoplasmic ratio. The nuclei are typically hyperchromatic, round to oval, and may appear angulated or slightly spindled, with finely granular chromatin and absent or inconspicuous nucleoli [32].

Several morphological features strongly support the diagnosis. These include nuclear molding, brisk mitotic activity, abundant apoptotic bodies, extensive geographic necrosis, and frequent crush artifact. Crush artifact refers to mechanical distortion of tumor cells during biopsy acquisition or processing, resulting in compressed cellular architecture, smudged chromatin, and loss of nuclear detail; in SCNEC, it may obscure diagnostic morphology and simulate lymphoma or other poorly differentiated small round blue cell tumors [24,32].

Another important histopathological aspect is the frequent occurrence of mixed tumors. In some cases, SCNEC coexists with squamous cell carcinoma, adenocarcinoma, or, more rarely, LCNEC. Recognition of such mixed histology is important, as limited biopsy material may sample only one component, thereby leading to incomplete or potentially misleading classification [23,35].

### 4.3. Large Cell Neuroendocrine Carcinoma

LCNEC of the cervix is less common than SCNEC but shares its aggressive clinical behavior. In contrast to SCNEC, LCNEC is composed of larger polygonal tumor cells with more abundant cytoplasm, vesicular or coarse chromatin, prominent nucleoli, and marked cytological atypia. The architectural pattern is often heterogeneous, with diffuse, organoid, trabecular, nested, insular, and rosette-like arrangements variably represented within the same tumor [36].

High mitotic activity and extensive necrosis are characteristic findings in LCNEC, supporting its classification as a poorly differentiated high-grade neuroendocrine carcinoma. Despite these features, the diagnosis may be challenging, particularly in fragmented or superficially sampled tissue, where LCNEC may mimic poorly differentiated adenocarcinoma, undifferentiated carcinoma, or non-neuroendocrine high-grade cervical malignancies. As in SCNEC, mixed tumors may also occur, and careful histological examination is required to identify neuroendocrine differentiation and to avoid under-recognition of additional tumor components [5,32].

### 4.4. Practical Histopathological Considerations

In routine practice, the histological suspicion of NECC should be raised by a combination of high-grade cytology and neuroendocrine-type architectural or cytological features. In SCNEC, these include diffuse sheets or nests of small to intermediate-sized cells, scant cytoplasm, nuclear molding, finely granular chromatin, brisk mitotic activity, abundant apoptosis, necrosis, and frequent crush artifact. In LCNEC, suspicion is supported by larger pleomorphic cells, more abundant cytoplasm, prominent nucleoli, organoid or trabecular growth, high mitotic activity, and necrosis.

Morphology alone is often insufficient for definitive diagnosis, particularly in small biopsies, crushed specimens, necrotic tumors, and mixed lesions. Therefore, cases with suggestive morphology should proceed to confirmatory immunohistochemical evaluation, as discussed in the following section.

## 5. Immunohistochemical Profile of Cervical Neuroendocrine Carcinoma

Immunohistochemistry is central to the diagnosis of NECC, particularly in limited biopsy specimens, tumors with crush artifact, and cases showing morphological overlap with other poorly differentiated cervical malignancies. In routine practice, the diagnosis should be based on the integration of histological appearance with a carefully selected immunohistochemical panel that confirms both neuroendocrine differentiation and epithelial lineage, while also supporting site-specific interpretation in the appropriate clinical context [1,3].

**Figure 1:**
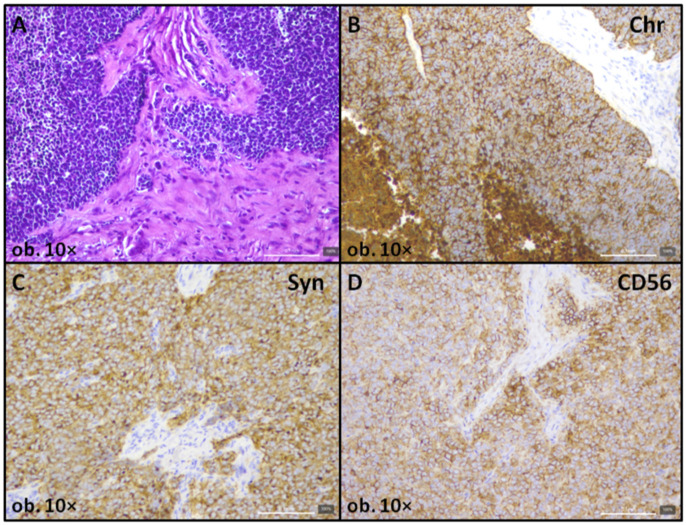
Neuroendocrine carcinoma of the uterine cervix: infiltrative malignant tumor composed of densely packed atypical cells, with scant cytoplasm and hyperchromatic nuclei (H&E staining) (**A**). Immunohistochemical staining demonstrates expression of neuroendocrine markers, including chromogranin (Chr) (**B**), synaptophysin (Syn) (**C**) and CD 56 (**D**).

The most widely used conventional neuroendocrine markers are synaptophysin, chromogranin A, CD56, and, less commonly, neuron-specific enolase. Synaptophysin is generally sensitive and often shows cytoplasmic staining in high-grade neuroendocrine carcinomas, whereas chromogranin A is more specific but may be focal or negative in poorly differentiated tumors. CD56 is sensitive but less specific, as it may be expressed in several non-neuroendocrine malignancies (Figure 1) [1,2,7].

In practical terms, no single neuroendocrine marker is sufficient in all cases. NECC is best supported by compatible morphology together with expression of more than one neuroendocrine marker and confirmation of epithelial differentiation. This is particularly important in SCNEC, where marker expression may be focal, weak, or occasionally absent despite classic morphology. Conversely, focal neuroendocrine marker expression in an otherwise typical non-neuroendocrine carcinoma should not be overinterpreted [2,3].

A critical and sometimes underemphasized step in the immunohistochemical evaluation is confirmation of epithelial differentiation. Broad-spectrum cytokeratins, including AE1/AE3 and CAM5.2, as well as low-molecular-weight keratins such as CK8/18, are useful for establishing epithelial lineage and for distinguishing neuroendocrine carcinoma from other small round blue cell tumors that may involve the cervix. This point is particularly relevant in tumors with scant cytoplasm, extensive necrosis, or crush artifact, in which morphology alone may be misleading. Although many NECC are keratin-positive, staining may be focal in some poorly differentiated tumors; therefore, a negative or limited keratin signal should be interpreted with caution and in conjunction with the broader immunophenotypic profile [1].

Among the newer markers, insulinoma-associated protein 1 (INSM1) has emerged as a highly sensitive indicator of neuroendocrine differentiation and appears especially useful in high-grade neuroendocrine carcinomas. Its expression has been reported in a high proportion of cases and may exceed the sensitivity of some conventional markers, making it a valuable adjunct in diagnostically challenging specimens. INSM1 is particularly useful when chromogranin A is negative or only weakly expressed, and when morphology raises suspicion for neuroendocrine carcinoma despite an incomplete conventional marker profile. Its nuclear staining pattern may also facilitate interpretation in crushed or necrotic biopsy material [2,12,13].

The proliferation marker Ki-67 may further support the diagnosis and biological interpretation of NECC. High Ki-67 labeling indices are typically observed in poorly differentiated neuroendocrine carcinomas and are consistent with their aggressive clinical behavior. Although Ki-67 is not specific for neuroendocrine differentiation, it can provide useful complementary information in the distinction between high-grade carcinoma and lower-grade neuroendocrine lesions, and it may also have prognostic and therapeutic relevance. In general, a markedly elevated proliferation index supports the diagnosis of a high-grade neuroendocrine carcinoma in the appropriate morphological and immunophenotypic setting [37,38,39,40].

Markers related to HPV-associated cervical carcinogenesis also have important interpretive value. Diffuse block-type p16 expression, defined as strong and continuous nuclear and cytoplasmic staining involving most tumor cells, is commonly observed in both SCNEC and LCNEC and reflects the strong association of these tumors with transforming high-risk HPV infection. However, p16 is not specific for neuroendocrine differentiation and may also be diffusely positive in other HPV-related cervical malignancies, including squamous cell carcinoma and usual-type adenocarcinoma. Therefore, p16 should be interpreted as a marker supporting cervical HPV-related origin rather than as evidence of neuroendocrine phenotype per se [39,41].

Altered p53 expression has also been described in NECC, reflecting underlying abnormalities in tumor suppressor pathways. Although p53 immunohistochemistry may have biological and, in some contexts, prognostic relevance, it is not a lineage-defining marker and should not be used as a primary tool for diagnostic classification. Similarly, expression of markers such as HER2/neu, EGFR, VEGF, cyclooxygenase-2, estrogen receptor, and progesterone receptor has been explored in selected studies, but these markers currently have more exploratory than routine diagnostic value in this setting [25,42].

One of the most important differential diagnostic issues is the distinction between primary cervical SCNEC and metastatic SCNEC from another site, particularly the lung. In this context, thyroid transcription factor-1 (TTF-1) requires careful interpretation. Although TTF-1 is classically associated with pulmonary small cell carcinoma, it may also be expressed in a subset of cervical SCNECs. Therefore, TTF-1 positivity does not exclude a primary cervical origin. Conversely, strong and diffuse TTF-1 expression, particularly in a patient with a smoking history, pulmonary mass, or radiological evidence of thoracic disease, should raise concern for metastatic pulmonary small cell carcinoma. Correlation with HPV-related markers, p16 staining pattern, clinical history, imaging findings, and the broader immunophenotypic profile is essential [1,43].

From a practical perspective, the immunohistochemical workup of a suspected NECC should ideally be tiered. In the first step, a core panel including synaptophysin, chromogranin A, CD56, p16, Ki-67, and broad-spectrum cytokeratins may be used to establish neuroendocrine differentiation, confirm epithelial lineage, and support an HPV-related cervical origin. In more challenging cases, especially when the initial panel yields equivocal results, additional markers such as INSM1, CK8/18, p40, p63, TTF-1, and selected site-specific markers may be incorporated to refine the differential diagnosis. This tiered approach is particularly useful in small biopsies and in tumors with mixed or ambiguous morphology [1,2,3,7]. Table 2 summarizes a practical immunohistochemical approach according to common diagnostic scenarios encountered in routine practice.

**Figure 2:**
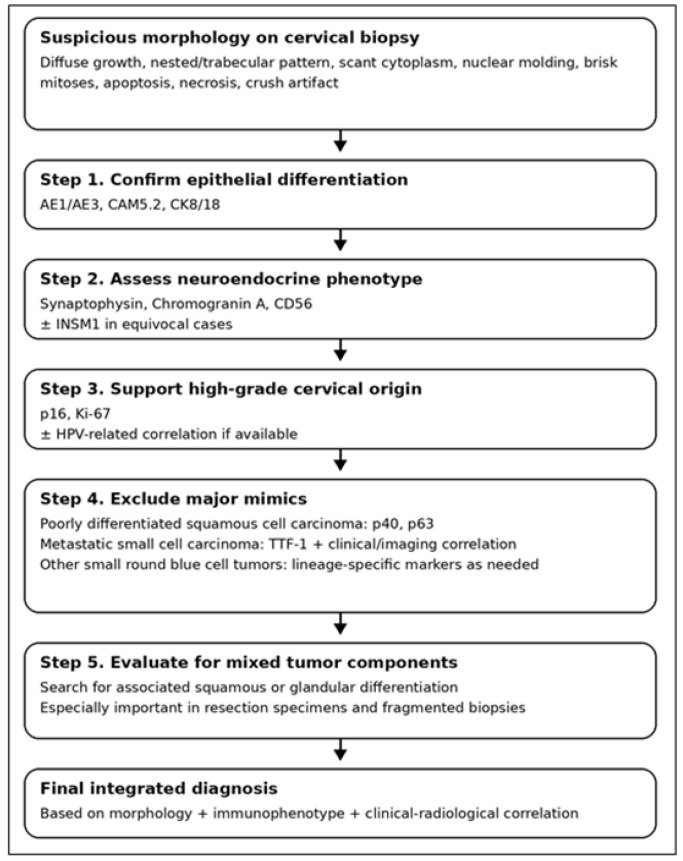
Practical diagnostic approach in suspected cervical neuroendocrine carcinoma. The proposed workflow outlines a stepwise diagnostic strategy for cases with morphological suspicion of cervical neuroendocrine carcinoma, particularly in small biopsies or poorly preserved specimens. The approach begins with recognition of suggestive histological features, including high-grade cytology, diffuse or nested growth, nuclear molding, necrosis, brisk mitotic activity, apoptosis, and crush artifact. Diagnostic confirmation requires demonstration of epithelial differentiation, usually by broad-spectrum cytokeratins, together with support for neuroendocrine differentiation using markers such as synaptophysin, chromogranin A, CD56, and INSM1. p16 expression and HPV testing may support an HPV-associated cervical origin, but should not be interpreted as evidence of neuroendocrine differentiation. In equivocal cases, additional markers and clinico-radiological correlation are required to exclude mimics, including poorly differentiated squamous cell carcinoma, adenocarcinoma, lymphoma, melanoma, sarcoma, and metastatic neuroendocrine carcinoma.

Overall, immunohistochemistry remains indispensable for the accurate classification of NECC. However, its greatest diagnostic value lies not in the isolated performance of individual markers, but in the combined interpretation of morphology, marker pattern, epithelial confirmation, HPV-related context, and clinico-radiological correlation. Such an integrated strategy is essential to minimize diagnostic error and to distinguish primary cervical neuroendocrine carcinoma from its major histological mimics. To facilitate routine diagnostic workup in morphologically suspicious cases, a practical stepwise diagnostic approach is summarized in Figure 2.

## 6. Diagnostic Pitfalls and Differential Diagnosis in Cervical Neuroendocrine Carcinoma

NECC is one of the most diagnostically challenging malignant tumors encountered in gynecologic pathology. The difficulty reflects its rarity, broad morphological spectrum, overlap with other poorly differentiated cervical and metastatic tumors, variable neuroendocrine marker expression, and frequent limitations of small or artifact-distorted biopsy samples. As a result, underdiagnosis and misclassification remain important risks in routine practice [2,44].

One of the most common diagnostic pitfalls is morphological overlap with other high-grade cervical malignancies. SCNEC may closely resemble poorly differentiated squamous cell carcinoma because both entities may show basaloid features, hyperchromatic nuclei, high nuclear-to-cytoplasmic ratios, extensive necrosis, and brisk mitotic activity. Likewise, LCNEC may be mistaken for poorly differentiated adenocarcinoma, undifferentiated carcinoma, or other non-neuroendocrine high-grade epithelial tumors, particularly when architectural neuroendocrine features are subtle or only partially represented. In these settings, morphology alone may be insufficient and should prompt a focused immunohistochemical workup [41,45].

Limited biopsy material is another major source of diagnostic error. Small cervical biopsies may not capture the full tumor spectrum, especially in mixed tumors, and crush artifact, necrosis, or fragmentation may obscure key nuclear and architectural features. In these settings, NECC may be mistaken for lymphoma, poorly differentiated squamous carcinoma, metastatic small cell carcinoma, or an undifferentiated malignant neoplasm. Awareness of this possibility is essential, because delayed recognition may affect staging and treatment planning [45,46,47].

Immunohistochemical interpretation can also be misleading when markers are considered in isolation. CD56 lacks specificity, chromogranin A may be focal or negative in poorly differentiated tumors, and occasional NECCs may show limited staining for conventional neuroendocrine markers. Conversely, focal neuroendocrine marker expression in a non-neuroendocrine carcinoma should not automatically lead to a diagnosis of NECC. A coherent diagnosis requires concordance between morphology, epithelial differentiation, neuroendocrine marker expression, HPV-related context, and clinical findings [1,2,3,7,41].

Distinguishing primary NECC from metastatic neuroendocrine carcinoma involving the cervix is particularly important. Metastatic pulmonary or gastrointestinal neuroendocrine carcinomas may closely mimic primary cervical tumors. TTF-1 has limited discriminatory value because it may be positive in both pulmonary small cell carcinoma and a subset of cervical SCNECs; however, strong and diffuse TTF-1 expression in a patient with a smoking history or pulmonary lesion should raise concern for metastatic pulmonary origin. Accurate classification requires correlation with clinical history, imaging, HPV testing, p16 pattern, and the overall immunophenotype [1,7,43,45].

Mixed tumors represent another frequent diagnostic challenge. NECC may coexist with squamous cell carcinoma, adenocarcinoma, or, more rarely, another neuroendocrine component. Limited biopsy may sample only the non-neuroendocrine component, causing the NECC component to be missed, or only the neuroendocrine component, obscuring the mixed nature of the lesion. Thorough sampling and component-specific immunohistochemistry are therefore essential, particularly in resection specimens and fragmented biopsies [1,47].

The differential diagnosis also includes non-epithelial and non-cervical entities, particularly in tumors composed of small round blue cells. Lymphoma, melanoma, sarcoma, and other primitive-appearing malignancies may mimic NECC when keratin staining is weak or morphology is compromised by artifact. Confirmation of epithelial differentiation is therefore a critical first step, followed by lineage-specific markers when hematolymphoid, melanocytic, or mesenchymal tumors are considered. The main differential diagnostic features are summarized in Table 3 [1,32,41,46,47].

**Figure 3:**
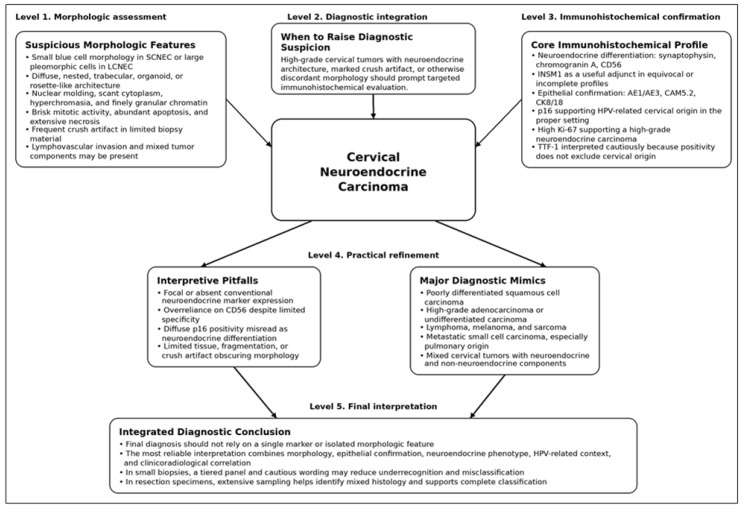
Integrated morphologic, immunohistochemical, and differential diagnostic framework for cervical neuroendocrine carcinoma. This schematic summarizes the main diagnostic levels that should be integrated when evaluating a suspected cervical neuroendocrine carcinoma. Morphological assessment should identify features suggestive of SCNEC or LCNEC, including high-grade cytology, neuroendocrine-type architecture, necrosis, high mitotic activity, and crush artifact. Immunohistochemical evaluation should confirm epithelial lineage and neuroendocrine differentiation while avoiding overinterpretation of isolated marker expression. HPV-related findings, including diffuse block-type p16 expression and high-risk HPV detection, may support cervical origin but are not specific for neuroendocrine phenotype. The framework also highlights major diagnostic pitfalls, including limited biopsy material, mixed tumors, focal neuroendocrine marker expression in non-neuroendocrine carcinomas, and metastatic neuroendocrine carcinoma involving the cervix. Final classification should rely on integrated interpretation of morphology, immunophenotype, HPV-related context, clinical history, and imaging findings.

From a practical perspective, the most reliable way to reduce diagnostic error is to adopt a structured interpretive approach. In tumors showing diffuse growth, nested or trabecular architecture, scant cytoplasm, brisk mitotic activity, necrosis, or crush artifact, NECC should be considered early in the differential diagnosis and followed by a focused immunohistochemical panel including epithelial and neuroendocrine markers [8,10]. In equivocal cases, second-line markers may help refine classification, but final interpretation should always integrate morphology, immunophenotype, HPV-related context, and clinico-radiological findings, particularly when metastatic disease or mixed histology is suspected. Such an approach is especially valuable in small biopsies, where limited tissue and artifact may otherwise lead to under-recognition or misclassification [16,44]. To further integrate the main morphologic, immunohistochemical, and differential diagnostic considerations relevant to this entity, a multilevel practical framework is summarized in Figure 3.

## 7. Prognostic Factors and Therapeutic Considerations in Cervical Neuroendocrine Carcinoma

NECC, particularly SCNEC, is associated with an aggressive clinical course and substantially poorer outcomes than conventional cervical squamous cell carcinoma and adenocarcinoma, with early lymphovascular invasion, frequent nodal and distant metastatic spread, high recurrence risk, and reduced long-term survival. Reported 5-year overall survival varies widely according to stage and cohort composition, but remains poor, with all-stage 5-year survival commonly reported in the range of approximately 25–40% for SCNEC. In some series, early-stage disease has better outcomes than advanced-stage disease, whereas patients with distant metastasis or stage IV disease have very limited long-term survival. These data should be interpreted cautiously because most studies are retrospective and include small, heterogeneous cohorts [48,49,50].

Among the clinicopathological variables examined in the literature, FIGO stage remains the most consistently validated prognostic factor. As in other cervical malignancies, increasing stage reflects greater local extension and metastatic spread; however, in neuroendocrine carcinoma, the prognostic impact of stage is particularly pronounced. Patients with disease confined to the cervix generally have better outcomes than those with parametrial extension, nodal disease, or distant metastases, although survival remains lower than expected for stage-matched non-neuroendocrine cervical carcinomas. Advanced-stage disease, especially stage IVB, is associated with extremely poor survival [25,49,50].

Lymph node involvement is also an important adverse prognostic feature and has been associated with reduced disease-free and overall survival in several studies. Additional parameters reported to correlate with poorer outcomes include large tumor size, deep stromal invasion, parametrial extension, positive surgical margins, and, in some series, chromogranin A expression. However, because most published data are derived from retrospective cohorts and small institutional series, the independent prognostic value of many of these variables remains difficult to define with certainty. Some studies have not confirmed statistically significant associations for factors such as age at diagnosis, tumor size, or specific treatment modalities, highlighting the heterogeneity and limited statistical power of the available evidence [51,52].

Because prospective NECC-specific treatment trials are lacking, current therapeutic recommendations are based mainly on retrospective series, expert consensus, and extrapolation from pulmonary small cell carcinoma and conventional cervical cancer. Therefore, treatment statements should be interpreted as practical considerations rather than evidence-based guidelines. Nevertheless, there is broad agreement that high-grade NECC should not be treated in the same manner as usual cervical squamous cell carcinoma or adenocarcinoma, and that systemic therapy plays a central role even in apparently localized disease [53]. In practical terms, management is usually stage-oriented. For apparently localized early-stage disease, radical surgery with nodal assessment may be considered in selected patients, but systemic platinum–etoposide-based chemotherapy is generally incorporated because of the high risk of occult dissemination. For locally advanced or unresectable disease, concurrent chemoradiotherapy with a platinum-based regimen is commonly used. For metastatic or recurrent disease, systemic therapy remains the mainstay, and molecular profiling may be considered to identify potentially actionable alterations or clinical trial options, although targeted and immune-based approaches remain investigational.

In early-stage tumors, particularly those measuring 4 cm or less and lacking radiological evidence of nodal involvement, radical hysterectomy with pelvic lymphadenectomy is often considered, usually followed by adjuvant chemotherapy. Platinum-based regimens combined with etoposide remain the most commonly employed systemic treatment backbone, reflecting the biological and therapeutic parallels with SCNEC at other sites. In selected patients, adjuvant radiotherapy may also be considered on the basis of pathological risk factors and multidisciplinary assessment. Although surgery continues to be incorporated in many treatment algorithms for limited-stage disease, surgery alone is generally considered inadequate for high-grade NECC [50,54]. However, the optimal sequencing of surgery, chemotherapy, and radiotherapy remains uncertain.

For locally advanced disease, unresectable tumors, or patients who are not appropriate surgical candidates, concurrent chemoradiotherapy is generally regarded as the standard therapeutic approach. In this setting, platinum–etoposide combinations remain widely used, while carboplatin may be substituted for cisplatin when clinically indicated. Other regimens have been described, including combinations involving paclitaxel, irinotecan, or older multiagent protocols, but the supporting evidence is limited and no universally accepted regimen has been established [8,51]. Comparative evidence among chemotherapy regimens remains limited.

An important clinical point is that the number and intensity of systemic treatment cycles may influence the outcome. Retrospective data have suggested improved recurrence-free survival in patients receiving more sustained platinum–etoposide-based adjuvant therapy, but these findings are vulnerable to selection bias and should not be interpreted as definitive. Immunotherapy and molecularly guided targeted treatment remain investigational in NECC; they may be considered in selected recurrent or metastatic cases after molecular profiling, preferably within clinical trials or multidisciplinary molecular tumor board discussion [51,52,53].

Fertility-sparing management remains highly controversial and is generally not recommended in high-grade NECC. Although isolated cases have been reported, the aggressive nature of the disease and the high risk of early dissemination make conservative treatment strategies difficult to justify outside highly selected and exceptional circumstances. In most cases, oncologic safety takes precedence over fertility preservation [55,56].

Overall, NECC should be approached as an aggressive cervical malignancy with early systemic potential. Prognosis is driven primarily by stage, nodal status, metastatic burden, and treatment feasibility, while management generally requires multidisciplinary planning and combined-modality therapy. Although molecular profiling may identify potentially actionable alterations in selected cases, no molecular marker or targeted therapy has yet been validated for routine treatment selection. Future multicenter studies are needed to define optimal stage-specific treatment strategies and integrate molecular findings into clinically useful prognostic and therapeutic models.

## 8. Conclusions and Future Perspectives

NECC is a rare but highly aggressive malignancy that continues to represent a major diagnostic challenge in routine gynecologic pathology. Its low incidence, marked morphological overlap with other poorly differentiated cervical and metastatic tumors, frequent presentation in limited biopsy material, and occasional variability in neuroendocrine marker expression all contribute to the risk of under-recognition or misclassification. In this setting, accurate diagnosis is essential, as it has direct implications for staging, therapeutic decision-making, and overall patient management [3,9,57].

The currently available evidence supports a diagnostic approach based on the integration of morphology, immunohistochemistry, and clinico-radiological correlation. Histological evaluation remains the foundation of diagnosis, but it is most reliable when complemented by confirmation of epithelial differentiation, appropriate assessment of neuroendocrine phenotype, and careful consideration of HPV-associated cervical origin. This integrated strategy is particularly important in diagnostically challenging scenarios, including small biopsies with crush artifact, tumors with mixed histology, and cases in which metastatic neuroendocrine carcinoma must be excluded. In this context, structured immunohistochemical panels and clinico-radiological correlation remain more immediately applicable than any single molecular marker [20,21,22,58].

At the biological level, recent studies have improved the understanding of NECC by highlighting its strong association with high-risk human papillomavirus, especially HPV18, as well as recurrent molecular alterations involving pathways related to cell proliferation, survival, and genomic instability. Although these findings have not yet been fully incorporated into routine diagnostic algorithms or standard therapeutic stratification, they reinforce the concept that NECC represents a biologically distinctive subset of cervical cancer with specific pathological and clinical features [22,28].

Despite these advances, important limitations remain. Most of the currently available data are derived from retrospective studies, small institutional series, and case reports, reflecting the rarity of the disease and the difficulty of assembling large homogeneous cohorts. As a result, diagnostic criteria are not always applied uniformly, the prognostic significance of several clinicopathological and molecular variables remains incompletely defined, and therapeutic recommendations continue to rely largely on extrapolation from other high-grade neuroendocrine malignancies. To date, single-cell RNA sequencing has not been established as a routine or widely reported approach in sizeable NECC cohorts. Future single-cell and spatial transcriptomic studies could clarify intratumoral heterogeneity, neuroendocrine lineage programs, epithelial–mesenchymal transition, immune microenvironment composition, and therapy-resistant subclones. Similarly, paired analyses of primary, recurrent, and metastatic lesions using WGS, RNA-seq, methylation profiling, and chromatin-accessibility assays could improve understanding of tumor evolution, metastatic dissemination, epigenetic regulation, and genomic instability in NECC.

Future experimental work should also prioritize NECC-specific preclinical models, including well-characterized cell lines, patient-derived organoids, and patient-derived xenografts. Such models would allow functional testing of platinum resistance, PI3K/AKT/mTOR and RAS/MAPK pathway dependence, HPV-driven transcriptional programs, immune-evasion mechanisms, and candidate targeted or immune-based therapies. Given the rarity of NECC, multicenter collaboration and shared biobanking will be essential for developing and validating these resources [10,12,20].

Overall, improving outcomes in NECC depends not only on therapeutic progress but also on earlier recognition, more consistent pathological classification, and a more precise understanding of tumor biology. In this regard, a multidisciplinary and diagnostically rigorous approach remains the most important foundation for future progress.

## Figures and Tables

**Table 1:** Summary of selected molecular profiling studies in cervical neuroendocrine carcinoma, highlighting the sequencing approach, cohort size, recurrent gene alterations, reported frequencies, and main study limitations.

Study	Year	Method/Cohort	No. of Cases	Main Reported Molecular Findings	Interpretation/Limitation
Xing et al. [28]	2018	Targeted NGS using a 637-gene panel in cervical SCNEC	10	Driver mutations in 8/10 tumors. Main recurrent alterations: TP53 4/10, 40%; PIK3CA 3/10, 30%. Additional altered genes included KRAS, ERBB2, c-MYC, NOTCH1, BCL6, NCOA3, PTEN, RB1, BRCA1/2, ARID1B.	Small sequencing study; supports involvement of MAPK, PI3K/AKT/mTOR, and TP53/BRCA-related pathways.
Hillman et al. [27]	2020	WES of fresh-frozen high-grade NECC with matched normal tissue	15	PIK3CA p.E545K 4/15, 27%; KRAS/GNAS 2/15, 13%; TP53 2/15, 13%; PTEN loss 5/15, 33%; PI3K or MAPK pathway activating alterations in 67%.	WES-based comparative genomic study; suggests NECC may be genetically closer to common cervical cancers than to pulmonary/bladder small cell carcinoma.
Cimic et al. [16]	2021	IHC, DNA/RNA NGS, NTRK fusion testing, MSI, TMB, and immune-oncology biomarkers	62	Most frequent pathogenic mutations: PIK3CA 11/62, 17%; TP53 11/62, 17%; KRAS 7/62, 11%; PTEN 6/62, 10%; CTNNB1 3/62, 5%. No MSI-high tumors were identified.	Shows limited but present targetable biomarkers; frequent DLL3 expression but low PD-L1 positivity and low TMB-high frequency.
Pei et al. [31]	2021	520 cancer-related gene NGS panel in surgically treated cervical SCNEC	51	Somatic genomic alterations in 50/51, 98%. Main mutations: TP53 12.24%, KRAS 10.20%, PIK3CA 10.20%, KMT2D 8.16%, PTEN/ATM/ATRX/PRKDC 6.12% each. Main amplifications: MYC 14.29%, IRS2 14.29%, TERT 12.24%, RICTOR 10.20%, SOX2 6.12%.	Larger SCNEC-specific NGS cohort; two cases were MSI-H/dMMR and p53-pathway alterations were associated with poorer 3-year OS.

**Table 2:** Practical immunohistochemical approach in suspected cervical neuroendocrine carcinoma.

Diagnostic Scenario	First-Line Markers	Additional Markers/Tests	Practical Interpretation
Classic morphology suspicious for SCNEC or LCNEC	AE1/AE3 or CAM5.2; synaptophysin; chromogranin A; CD56; INSM1; Ki-67; p16	HPV ISH or PCR when site confirmation is needed	Diagnosis is supported by compatible morphology, epithelial differentiation, more than one neuroendocrine marker, high proliferation index, and HPV-associated context.
Limited biopsy or crush artifact	AE1/AE3; CAM5.2; INSM1; synaptophysin; CD56; Ki-67	p40/p63; LCA/CD45; SOX10/S100; desmin/myogenin according to morphology	Confirms epithelial and neuroendocrine differentiation while excluding lymphoma, melanoma, sarcoma, and poorly differentiated squamous carcinoma.
Poorly differentiated squamous carcinoma mimic	p40; p63; AE1/AE3; synaptophysin; chromogranin A; CD56; INSM1; p16	HPV testing if needed	Diffuse squamous marker expression with absent or only focal neuroendocrine staining favors poorly differentiated squamous carcinoma.
Poorly differentiated adenocarcinoma or undifferentiated carcinoma mimic	AE1/AE3; CK7; neuroendocrine markers; INSM1; Ki-67; p16	CEA, PAX8, ER/PR, or site-specific markers as clinically indicated	Isolated focal neuroendocrine marker expression is insufficient for NECC without supportive morphology.
Possible metastatic pulmonary small cell carcinoma	TTF-1; neuroendocrine markers; AE1/AE3; p16	HPV ISH/PCR; thoracic imaging; clinical history	TTF-1 positivity alone cannot assign pulmonary origin; strong diffuse TTF-1 with smoking history or lung mass favors metastasis.
Suspected mixed cervical tumor	Broad sampling; AE1/AE3; neuroendocrine markers; INSM1; p40/p63; glandular markers; p16	HPV testing; component-specific staining	Each component should be documented, because limited biopsy may sample only one component.

**Table 3:** Summarized differential diagnosis of neuroendocrine carcinoma of the cervix (NECC) and its major mimics. SCNEC, small cell neuroendocrine carcinoma; LCNEC, large cell neuroendocrine carcinoma; SCC, squamous cell carcinoma; NEN, neuroendocrine neoplasm; IHC, immunohistochemistry.

Entity	Key Morphology	Useful Markers	Main Pitfall	Practical Clue
SCNEC	Small blue cells, scant cytoplasm, nuclear molding, necrosis, crush artifact	Synaptophysin, CD56, chromogranin A, INSM1, cytokeratins, p16, Ki-67	Mimics poorly differentiated SCC, lymphoma, or metastatic small cell carcinoma	Confirm epithelial + neuroendocrine differentiation; correlate with imaging
LCNEC	Large pleomorphic cells, prominent nucleoli, organoid/trabecular growth, necrosis	Synaptophysin, CD56, chromogranin A, INSM1, cytokeratins, p16, Ki-67	Mimics high-grade adenocarcinoma or undifferentiated carcinoma	Look for neuroendocrine architecture and supportive IHC
Poorly differentiated SCC	Basaloid/solid growth, atypia, necrosis	p40, p63, cytokeratins, p16	Focal neuroendocrine staining may mislead	Diffuse p40/p63 favors SCC
High-grade adenocarcinoma	Glandular, cribriform, papillary, or solid growth	Cytokeratins, CK7, CEA, p16; neuroendocrine markers usually absent/focal	May mimic LCNEC	Isolated focal neuroendocrine staining is insufficient for NECC
Undifferentiated carcinoma	Solid high-grade tumor without clear lineage	Cytokeratins; lineage markers as needed	NECC may be missed in necrotic tissue	Add neuroendocrine markers if morphology is suspicious
Lymphoma	Discohesive small round blue cells	CD45 and lineage-specific hematolymphoid markers	Crushed biopsy may mimic SCNEC	Cytokeratin negativity favors lymphoma
Melanoma	Discohesive/epithelioid cells, pigmentation variable	SOX10, S100, melanocytic markers	Amelanotic melanoma may mimic carcinoma	Consider if cytokeratins are negative
Sarcoma/mesenchymal tumor	Spindle, pleomorphic, or primitive cells	Mesenchymal markers; cytokeratins usually negative	May simulate poorly differentiated carcinoma	Lack of epithelial markers prompts mesenchymal workup
Metastatic pulmonary small cell carcinoma	Similar to cervical SCNEC	TTF-1, neuroendocrine markers, p16, HPV testing	TTF-1 overinterpretation	Lung lesion/smoking history supports pulmonary origin; HPV supports cervical origin
Metastatic GI/pancreatobiliary NEN	Secondary cervical involvement	Neuroendocrine markers; site-specific markers	May be misclassified as primary cervical NECC	Clinical history and imaging are essential
Mixed cervical tumor	NECC with squamous or glandular component	Component-specific IHC	One component may be missed	Sample extensively

## Data Availability

No new data were created or analyzed in this study. Data sharing is not applicable to this article.
